# Supplementary material for: CSI: Contrastive data Stratification for Interaction prediction and its application to compound–protein interaction prediction
Source: Bioinformatics. 2023 Jul 25;39(8):btad456. doi: 10.1093/bioinformatics/btad456 (PMC10423023; doi:10.1093/bioinformatics/btad456)
Supplement: btad456_Supplementary_Data [file btad456_supplementary_data.zip › CSI_Bioinformatics_Supplementary_final.pdf]

CSI: **C**ontrastive Data **S**tratification for **I**nteraction Prediction and its Application to Compound-Protein Interaction Prediction
Supplementary File

Apurva Kalia[1], Dilip Krishnan[2], and Soha Hassoun[1,3]

[1]Department of Computer Science, Tufts University, Medford, MA 02155, USA
[2]Google Research
[3]Department of Chemical and Biological Engineering, Tufts University, Medford, MA 02155, USA.

S1 Details of Baseline Model

Our baseline model is based on GraphDTA (Nguyen *et al.*, 2021). GraphDTA offers a simple and generalizable method to create graph-based encoders for molecules represented in SMILES and CNN- based encoders for sequences and achieves 10-15% improved results compared to other models (Öztürk *et al.*, 2018, 2019; He, 2016; Cichonska *et al.*, 2017), and thus is a strong baseline. The baseline model architecture comprises of encoders for molecules and sequences, followed by MLP layers for interaction prediction (Figure S1). Compounds represented in SMILES format are converted to a molecular graph and processed using Graph Convolutional Networks. For protein sequences, each amino acid within protein sequences (in FASTA format) is first converted to a numeric code used to generate learnable embeddings. Sequence embeddings are passed to a protein encoder which consists of a 1D Convolutional Neural Network (CNN) followed by a pooling layer and a fully connected layer. Protein representations in FASTA format do not capture connection information between amino acids. A 1D CNN is suited for this sequence-like format. The compound and sequence embeddings are concatenated for the final interaction likelihood prediction. The final predictor is a 3-layer MLP with the first two layers each reducing the embedding dimensionality by half and the final layer making a binary prediction. Importantly, the architecture of the GCN and CNN encoders of the baseline model are used for CSI to ensure a fair comparison between the CSI and baseline model.

S2 Metrics used for model performance

To measure the model performance, it is important to choose metrics that can measure the model's ability to discriminate between positive and negative examples without needing to define a threshold dividing the positive from negative cases. This is important because the threshold could vary for different molecule-enzyme combinations. We used the following metrics to measure model performance:

- **Average Precision (AP)** is measured across each dataset, reflecting the model's ability to distinguish positive and negative examples.
- **R-precision**, also measured across each dataset, measures the model's ability to accurately predict the R known positive interactions.
- **Mean Average Precision (MAP)** measures the AP per compound (or per protein sequence) averaged over interactions sorted by compound (or protein sequence), thus indicating the model's ability to predict the likelihood of interaction for a given compound (or protein sequence).

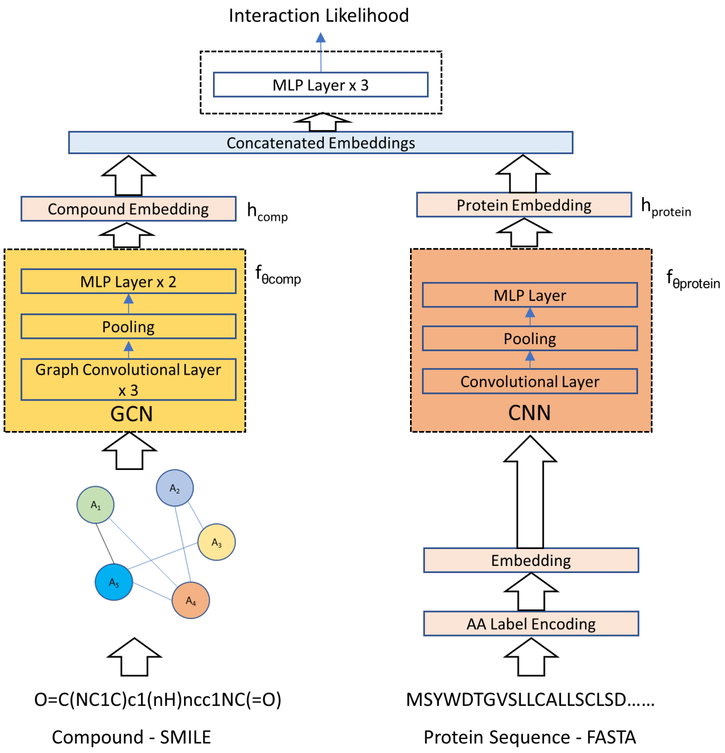

Figure S1: Compound-protein interaction prediction model used as the baseline. Interaction likelihood is predicted based on learned molecular and protein interactions.

- **MAP@3** reports MAP on the top 3 ranked items i.e the top 3 sequences per compound, or the top 3 compounds per sequence.
- **Precision@1** measures the ability of the model to correctly predict a top ranked interacting item.

Table S1: Statistics for the KEGG dataset for three different stratification strategies by interaction features. We report the total number of objects in each view with each stratification strategy, the average number of objects in each views over all keys, as well as the distribution of objects in each view.

	Number of views	Mean objects	std-dev objects	Max objects
(A) Stratification on reaction. Number of keys is 6,059				
V_1(comp-comp)	9,091	1.50	0.58	4
V_2(comp-seq)	124,046	20.47	35.32	387
V_3(seq-seq)	52,759	8.71	15.36	129
(B) Stratification on RCLASS. Number of keys is 2,158				
V_1(comp-comp)	5,765	2.67	7.09	134
V_2(comp-seq)	172,382	79.88	441.91	15635
V_3(seq-seq)	50,723	23.50	107.47	2994
(C) Stratification on EC. Number of keys is 3,363				
V_1(comp-comp)	8,493	2.53	3.00	79
V_2(comp-seq)	98,254	29.22	170.72	7500
V_3(seq-seq)	17,563	5.22	8.47	129

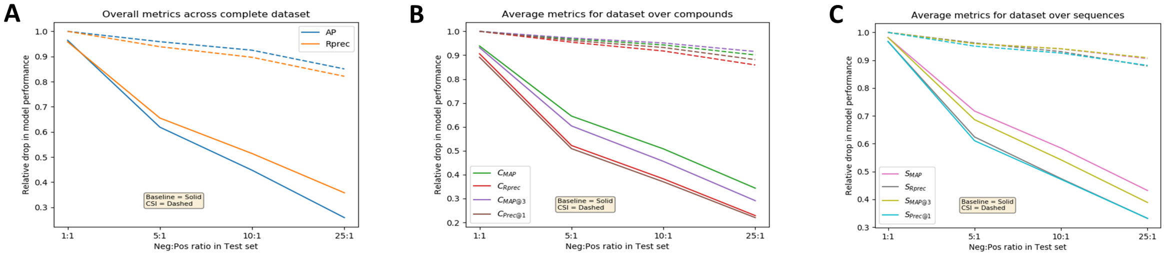

Figure S2: Model performance evaluation for various negative-to-positive ratios in the Test set. (A) AP and R-precision trends for various negative-to-positive ratios for Test set. (B) MAP, mean R-Precision, MAP@3, R-Precision@1 trends for Test set interactions sorted by compounds. (C) MAP, mean R-Precision, MAP@3, R-Precision@1 trends for Test set interactions sorted by sequences.

S3 Scaling of model performance with increasing negative to positive ratio

We measured model performance scaling with respect to the negative-to-positive ratio (Figure S2). Assuming the performance with 1:1 ratio to be 1.0, baseline model AP performance drops by 73% when using 25:1 ratio. Meanwhile, the performance of the CSI model drops only by 18% at the 25:1 ratio. For metrics measured per compound, the MAP metric drops by 75% for the baseline model for the 25:1 ratio whereas the same metric drops by only 14% for the CSI model. For metrics measured by sequence, the drops in MAP are 66% and 13% for the baseline and CSI model respectively. These results indicate that CSI performs better than the baseline model at predicting the negatives correctly. Further, the positives and negatives continue to be well separated even as the ratio of negatives increases.

S4 Details of KEGG stratification by reaction features

An enzymatic dataset like KEGG can also be stratified by reaction features - reaction, RCLASS and EC numbers. For each strata (Table S1), three different views of the data are possible: substrate-product pairs, compounds-sequence pairs and pairs of sequences. The number of keys per strategy differ, where stratification on reactions provides the most number of keys. The key choices subsequently affect the total number of views and the size of each strata within the views. Regardless of the key, there are more compound-sequence views (V_2) than the other two views, and fewest compound-compound views (V_1). We examine the strata to determine if any one particular reaction consistently contributed to the maximum strata size. The largest compound-sequence partition under the reaction stratification strategy is due to the ammonia-ubiquinol reaction (KEGG reaction R00148), which contributes to nitrogen metabolism. This reaction is catalyzed by ammonia mono-oxygenase (EC 1.14.99.39), which is present in 46 organisms leading to many sequences for the same enzyme. For RCLASS-based stratification, the largest compound-sequence partition is for RC00001 which is part of glutathione metabolism. This basic reaction class encompasses 14 different reactions, catalyzed by 18 different EC classes - leading to a large number of compound-sequence pairs. For EC-based classification, the largest compound-sequence partition is for glutathione transferase (EC 2.5.1.18), which catalyzes 24 reactions, and present in 423 different organisms.

For training, validation and testing on the interaction features, the methodology that was followed is similar to our prior strategy (Section 3.3 in the paper). Training, validation and test were split in 8:1:1 ratio. In addition, a test set with 5x more negatives than positives as well as the Unseen Test set were created.

References

Cichonska, A., Ravikumar, B., Parri, E., Timonen, S., Pahikkala, T., Airola, A., Wennerberg, K., Rousu, J., and Aittokallio, T. (2017). Computational-experimental approach to drug-target interaction mapping: a case study on kinase inhibitors. *PLoS computational biology*, **13**(8), e1005678.

He, T. (2016). *SimBoost: A Read-Across Approach for Drug-Target Interaction Prediction Using Gradient Boosting Machines*. Ph.D. thesis, Applied Sciences: School of Computing Science.

Nguyen, T., Le, H., Quinn, T. P., Nguyen, T., Le, T. D., and Venkatesh, S. (2021). Graphdta: Predicting drug–target binding affinity with graph neural networks. *Bioinformatics*, **37**(8), 1140–1147.

Öztürk, H., Özgür, A., and Ozkirimli, E. (2018). Deepdta: deep drug–target binding affinity prediction. *Bioinformatics*, **34**(17), i821–i829.

Öztürk, H., Ozkirimli, E., and Özgür, A. (2019). Widedta: prediction of drug-target binding affinity. *arXiv preprint arXiv:1902.04166*.
