# Supplementary figures and images for: CSI: Contrastive data Stratification for Interaction prediction and its application to compound–protein interaction prediction
Source: Bioinformatics. 2023 Jul 25;39(8):btad456. doi: 10.1093/bioinformatics/btad456 (PMC10423023; doi:10.1093/bioinformatics/btad456)

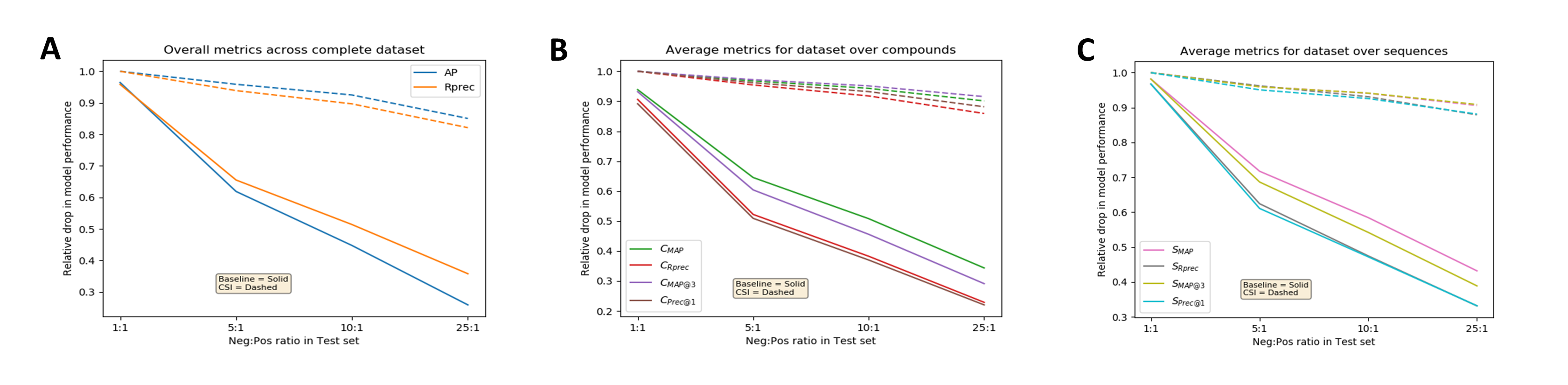

Supplement: btad456_Supplementary_Data [file btad456_supplementary_data.zip › CSI-Bioinformatics-Supplementary_final/figs/res_scale.png]

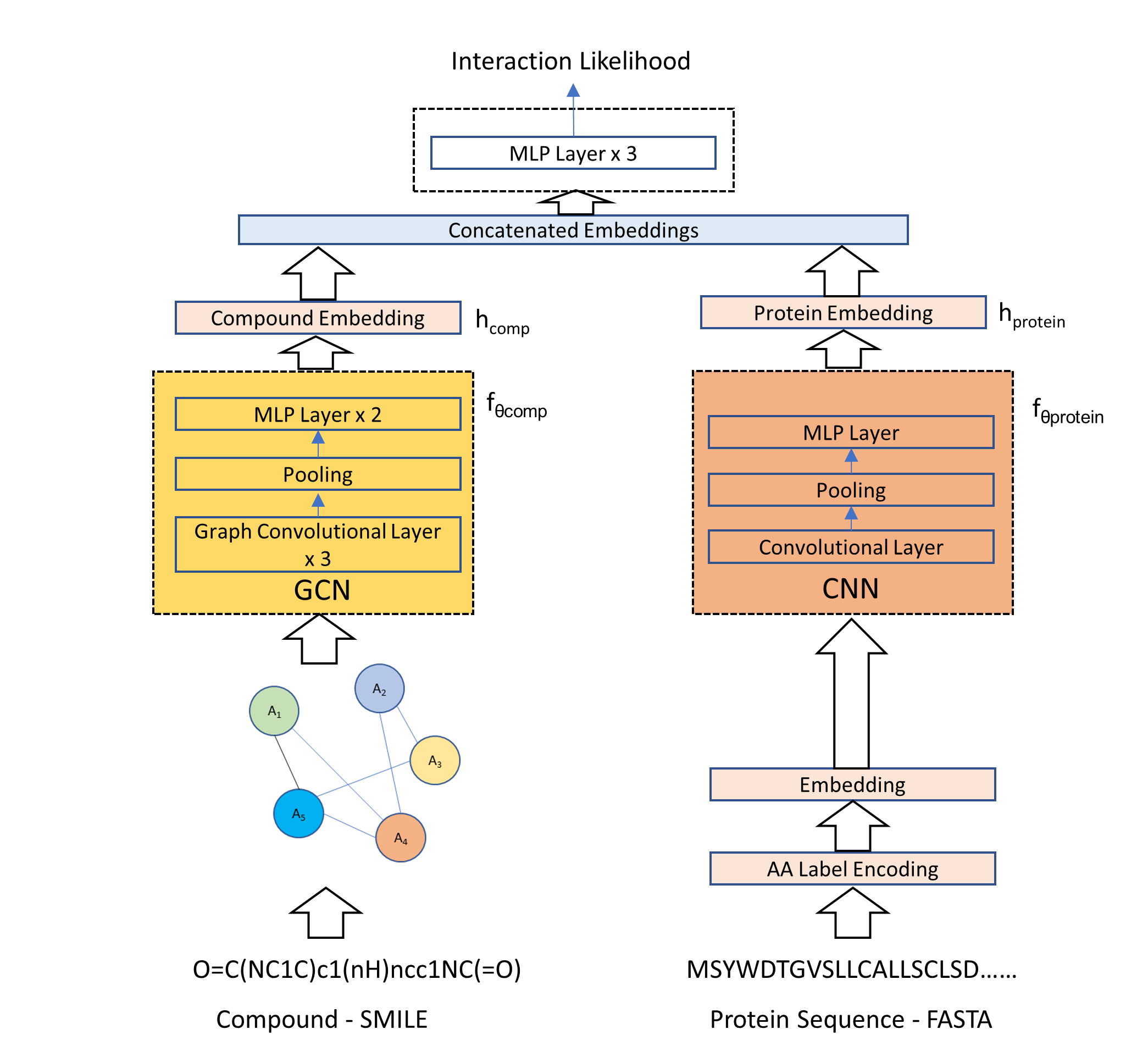

Supplement: btad456_Supplementary_Data [file btad456_supplementary_data.zip › CSI-Bioinformatics-Supplementary_final/figs/baseline.png]
